# Interleukin-1β Promotes Ox-LDL Uptake by Human Glomerular Mesangial Cells via LOX-1

**DOI:** 10.7150/ijms.43981

**Published:** 2020-04-27

**Authors:** Hua Liu, Yinping Li, Na Lin, Xingtong Dong, Wen Li, Yinghui Deng, Lina Ma

**Affiliations:** 1 Department of Nephrology, Xuanwu Hospital, Capital Medical University, Beijing 100053, China; 2 Department of Geriatrics, Xuanwu Hospital, Capital Medical University, Beijing 100053, China

**Keywords:** Interleukin-1β, oxidised low-density lipoprotein, lectin-like Ox-LDL receptor 1

## Abstract

The aim of this study was to determine whether interleukin-1β (IL-1β) promotes oxidised low-density lipoprotein (Ox-LDL) uptake by human glomerular mesangial cells (HMCs) and its effect on the expression of lectin-like Ox-LDL receptor 1 (LOX-1) and to identify pathways through which IL-1β affects lipid uptake. Confocal laser scanning microscopy and flow cytometry were used to observe the effect of IL-1β on lipid uptake by HMCs and the pathway by which IL-1β may mediate lipid uptake. Real-time polymerase chain reaction (PCR) and western blotting were used to evaluate the effect of IL-1β on LOX-1 expression. Confocal laser scanning microscopy and flow cytometry revealed that IL-1β promoted uptake of fluorescent Dil-labelled Ox-LDL(Dil-Ox-LDL) by HMCs and the enhanced uptake of Dil-Ox-LDL was partially inhibited by an anti-LOX-1 antibody evaluated by flow cytometry. Further, IL-1β promoted LOX-1 mRNA and protein expression of HMCs in a dose- and time-dependent manner. Thus, Ox-LDL is ingested by HMCs under basic conditions. Inflammatory cytokine IL-1β promotes Ox-LDL uptake by HMCs. Furthermore, IL-1β promotes the mRNA and protein expression of LOX-1, a specific receptor of Ox-LDL, suggesting that the enhancement of Ox-LDL uptake may be mediated by LOX-1 pathway. Anti-LOX-1 therapy may be a promising option for treatment of glomerulosclerosis.

## Introduction

Lipid metabolism abnormalities are often observed in many kidney diseases and hyperlipidaemia is a well-established risk factor for the progression of glomerulosclerosis 1. Present studies have demonstrated that glomerulosclerosis and atherosclerosis have similar pathological changes and pathophysiological mechanisms 2. Briefly, atherosclerosis is chronic inflammation of the vascular wall 3, and various kidney diseases are characterised by micro-inflammatory state 4. Inflammation together with lipids may participate in the occurrence and development of atherosclerosis and glomerulosclerosis 5. Therefore, studying how inflammation affects intracellular cholesterol homeostasis is key to understanding lipid-mediated kidney damage. Oxidised low-density lipoprotein (Ox-LDL) is an crucial risk factor for atherosclerosis and exerts its action through several different receptors, the most important of which is lectin-like oxidised low-density lipoprotein receptor 1 (LOX-1) in atherogenesis 6. There is growing focus on the influence of LOX-1 in inflammation related diseases, including coronary arterial disease, stroke, metabolic syndrome, and immunity diseases. Recently, LOX-1 is being viewed as a critical mediator of atherosclerosis and vascular inflammation. Elevated serum levels of soluble LOX-1, a fragment of the main LOX-1 molecule, have been evaluated as promising biomarkers for diagnosis and risk assessment of metabolic syndrome and coronary artery disease among the metabolic syndrome population 7.The present study on arthritis has demonstrated the roles of LOX-1/Ox-LDL system may be involved in the pathogenesis of arthritis by using LOX-1 knockout mice, additionally, LOX-1-positive synovial cells and chondrocytes may be potential therapeutic targets for arthritis prevention 8.The current review on regulation, signaling and various effects of LOX-1 has indicated the key contribution of LOX-1 to the atherogenic process. Anti-LOX-1 therapy has been shown to decrease inflammation, oxidative stress and atherosclerosis, making LOX-1 a promising target of therapy for atherosclerosis and related disorders 9. Studies of atherosclerosis have confirmed that many inflammatory cytokines can induce expression of LOX-1 in macrophages and endothelial cells 10,11. There are few studies on the role of LOX-1 in lipid uptake in HMCs, and the influence of inflammatory factors on LOX-1 expression has been scarcely studied. In this study, the effects of inflammatory cytokine interleukin-1β (IL-1β) on expression of LOX-1 in human glomerular mesangial cells (HMCs) were examined to explore the pathophysiological mechanism of inflammatory factors in lipid homeostasis at the cellular level in glomerulosclerosis.

## Materials and Methods

### Materials

RPMI-1640 culture solution, foetal calf serum (Gibco, Gaithersburg, MD, USA), Ox-LDL, HMCs (Basic Institute of Peking Union Medical College, Beijing, China), IL-1β, LOX-1 antibody (R&D, Abingdon, UK), and Dil-Ox-LDL (Biomed Technologies, Mt. Arlington, NJ, USA) were used. A polymerase chain reaction (PCR) system, reverse transcriptase buffer (Promega, Madison, WI, USA), dNTPs, and Oligo(dT) primers (Sangon Biotech, Shanghai, China) were used. The primer sequences were as follows: *LOX‑1* forward, 5'-ACAGAGGCCATTCCGAAATCA-3'; reverse, 5'-GGTAGAGTCTGGAGATGGACCACA-3'; *GAPDH* forward, 5′-GCACCGTCAAGGCTGAGAAC-3′; reverse, 5′-ATGGTGGTGAAGACGCCAGT -3′.

### Culture and identification of HMCs

HMCs were cultured in RPMI-1640 culture medium with 10% foetal calf serum (FCS) in an incubator with 5% CO2 at 37°C. HMCs were then washed with 0.01 M phosphate-buffered saline (PBS), digested with 0.25% trypsin/0.025% EDTA, subcultured by suspension in RPMI-1640 culture medium with 10% FCS. For the indirect immunofluorescence assay, cytoplasmic actin and collagen IV staining were positive, while cytokeratin and Factor VIII staining were negative.

### Confocal laser scanning microscopy

HMCs (5 × 10^4^/mL) were inoculated into 8-well plates, then cells were cultured to fusion and quiescent state. HMCs were randomly divided into the control group, IL-1β group (5 ng/mL IL-1β) and incubated for 12 hours, followed by 10 μg/mL Dil-Ox-LDL for continuous incubation for 5 hours. The culture plates were washed with PBS, then slides were fixed with 5% formalin and sealed with 90% glycerol. Under a confocal laser scanning microscope, the average fluorescence value per unit area of 30 cells was calculated after 6-8 fields were randomly selected per well.

### Flow cytometry analysis

Quiescent state HMCs were randomly divided into the control group, IL-1β group (5 ng/mL IL-1β), and LOX-1 receptor blocking+IL-1β group (2 ng/mL anti-LOX-1+5 ng/mL IL-1β; anti-LOX-1 antibody was applied 2 hours in advance) and incubated for 12 hours. Then, 10 μg/mL Dil-Ox-LDL was added to each group, followed by incubation for an additional 5 hours. After washing with PBS, digestion, centrifugation, repeated washing, and centrifugation, the cells were suspended in PBS, 6000 cells were counted by flow cytometry, and the average fluorescence value was calculated.

### Reverse transcription‑quantitative PCR (RT‑qPCR) analysis

After culturing to the quiescent state, HMCs were randomly divided into five groups, and the medium was replaced with a medium containing 5 ng/mL IL-1β. Cells were harvested at 0, 3, 6, 12, and 24 hours. Quiescent state cells were randomly divided into four groups. The cells were harvested after culturing in media containing 0, 2.5, 5, and 10 ng/mL IL-1β at 12 hours. Total RNA was extracted, and cDNA was synthesised. PCR amplification was performed in a 25-µL reaction system using the respective primers for 40-50 cycles at 95°C for 10 seconds, 95°C for 15 seconds, and 60°C for 30 seconds.

### Western blotting

After culturing to the quiescent state, HMCs were randomly divided into four groups, and the medium was replaced with medium containing 5 ng/mL IL-1β. Cells were harvested at 0, 8, 12, and 24 hours. Quiescent state cells were randomly divided into four groups. The cells were harvested at 12 hours after culturing in media containing 0, 2.5, 5, and 10 ng/mL IL-1β. Each lane of a gel was loaded with 250 μg of sample protein for electrophoresis. The protein was transferred to a membrane for 1 hour. After blocked with tris-buffered saline with Tween 20 (TBST)-bovine serum albumin (BSA) buffer overnight, the membrane was incubated with 0.2 ng/mL primary antibody by slowly shaking for 2 hours at 25°C, washed, incubated with 1:6000 secondary antibody by shaking for 1 hour, washed, and subjected to enhanced chemiluminescence (ECL) development.

### Statistical methods

SPSS21.0 was used for data analyses. Measurement data are expressed as means ± standard deviation (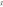 ± s). The* T2* test was used for pair-wise comparisons of mean values. One-way analysis of variance (ANOVA) was used for comparisons among multiple groups. P < 0.05 was considered statistically significant.

## Results

### Effect of IL-1β on Dil-Ox-LDL uptake by HMCs determined by confocal laser scanning microscopy

**Fig 1:**
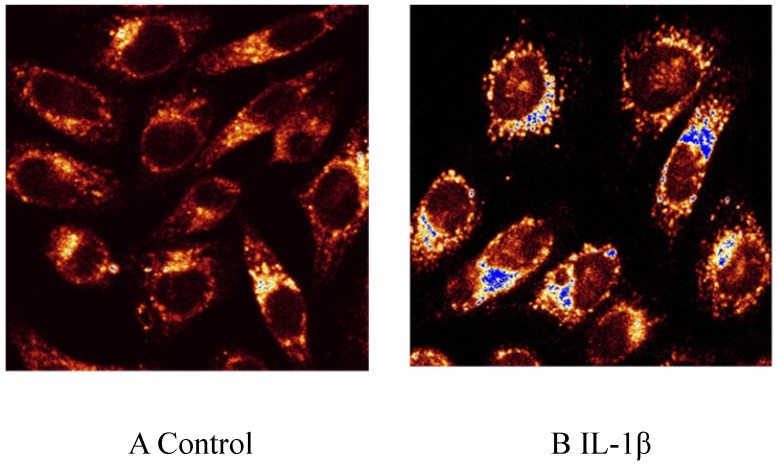
Effect of interleukin (IL)-1β treatment on uptake of Ox-LDL labeled with fluorescent Dil (Dil-Ox-LDL) by human mesangial cells for 12 hours. A: control; B: IL-1β.

HMCs in the control group internalised a small amount of Dil-Ox-LDL. 5 ng/mL IL-1β promoted uptake of Dil-Ox-LDL by HMCs; the intracellular fluorescence intensity was 4.95 times that of the control group after 12 hours (Fig. 1, magnification ×400).

### Flow cytometer counts

IL-1β promoted Dil-Ox-LDL uptake by HMCs. After 5 ng/mL IL-1β was used to stimulate HMCs for 12 hours, the intracellular fluorescence intensity was 5.06 times that of the control group. The anti-LOX-1 antibody was added 2 hours in advance. The average intracellular fluorescence value for the blocking group was reduced by 87.9% compared with that of the stimulation group (Table 1).

### Effect of IL-1β on LOX-1 mRNA expression

**Fig 2:**
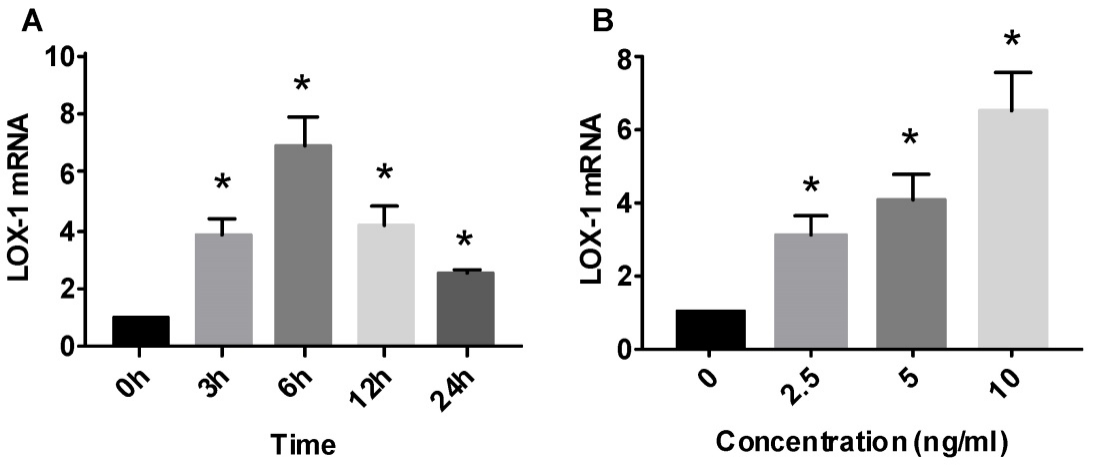
Effect of interleukin (IL)-1β treatment on lectin-like oxidized low-density lipoprotein receptor (LOX-1) mRNA expression in human mesangial cells. LOX-1 mRNA level expressed relative to that of GAPDH (n = 3).* P< 0.05

HMCs expressed *LOX-1* mRNA at basal state, and IL-1β promoted the expression of *LOX-1* mRNA in a time-dependent manner. IL-1β (5 ng/mL) was used to stimulate HMCs for 0-24 hours. LOX-1 levels began to increase (3.89 times) at 3 hours, reached a peak (6.87 times) at 6 hours, and gradually decreased thereafter. *LOX-1* mRNA levels at 24 hours were still higher (2.58 times) than those of the control group (Fig. 2).

IL-1β promoted *LOX-1* mRNA expression in a dose-dependent manner. IL-1β at 2.5, 5, and 10 ng/mL was used to stimulate HMCs for 12 hours. *LOX-1* mRNA in the 10 ng/mL group rose to a peak (6.57 times) (Fig. 2).

### Effect of IL-1β on LOX-1 protein expression

**Fig 3:**
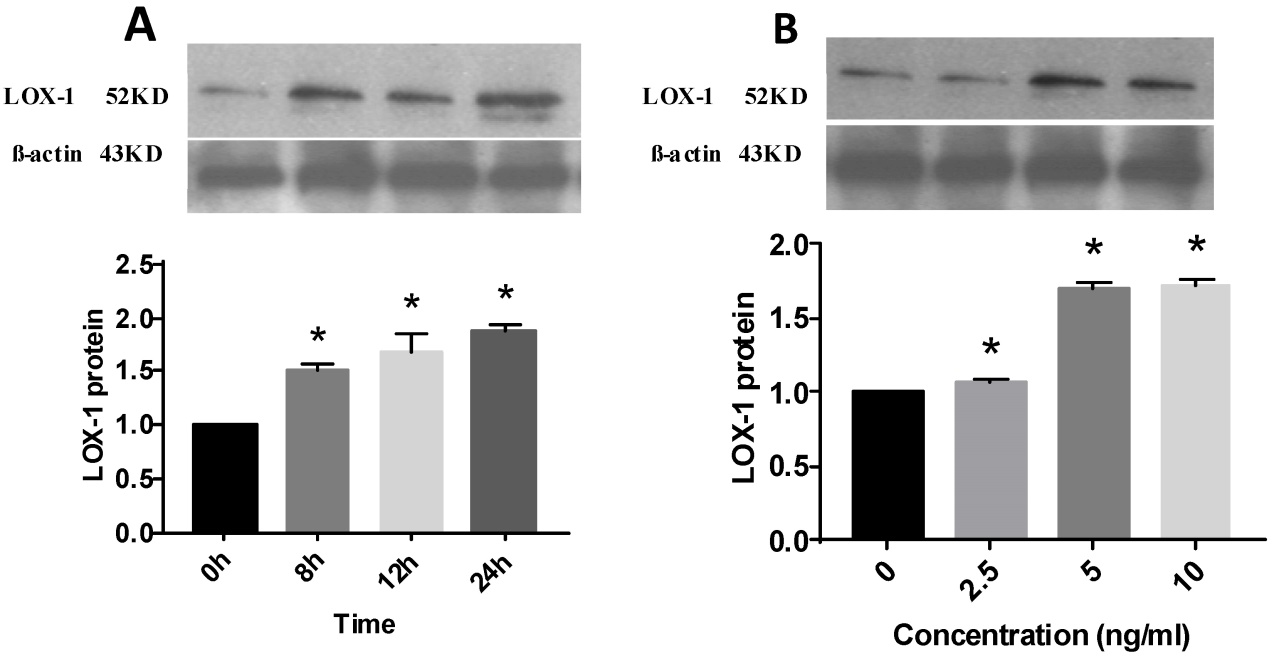
Effect of interleukin (IL)-1β treatment on lectin-like oxidized low-density lipoprotein receptor (LOX-1) protein expression in human mesangial cells. LOX-1 protein level expressed relative to that of β-actin (n = 3). * P< 0.05

IL-1β promoted the expression of LOX-1 in a time-dependent manner. IL-1β (5 ng/mL) was used to stimulate HMCs for 0-24 hours. A peak (1.88 times) was reached at 24 hours (Fig. 3).

IL-1β also promoted LOX-1 expression in a dose-dependent manner. IL-1β at 2.5, 5, and 10 ng/mL was used to stimulate HMCs for 12 hours. LOX-1 levels in the 10 ng/mL group were highest (1.72 times) (Fig. 3).

## Discussion

Clinical studies have demonstrated that hyperlipidaemia is associated with kidney diseases. In addition, hyperlipidaemia itself is an independent risk fator for the progression of renal injury. Increasing evidences have identified that glomerulosclerosis and atherosclerosis have similar pathophysiological mechanisms. Atherosclerosis has long been known to be a chronic inflammatory disease, and various kidney diseases, such as lupus nephritis and secondary Sjögren's syndrome, are characterized by a systemic inflammatory state 12. Therefore, it is believed that the joint action of inflammatory factors and lipids contributes to the pathogenesis of atherosclerosis and glomerulusclerosis. Furthermore, studying the mechanism by which inflammatory factors affect intracellular cholesterol homeostasis is crucial to clarify lipid-mediated renal injury.

### Inflammatory cytokine IL-1β promotes uptake of Ox-LDL by HMCs and affects cholesterol homeostasis at the cellular level

Present studies have showed that atherosclerosis is a chronic inflammatory disease of blood vessels that involves multiple cytokines 13. Ox-LDL is considered a key contributing factor of cardiovascular events and plays an important role in the occurrence and development of atherosclerosis 14. Ox-LDL binds to specific receptors and causes dysfunction of endothelial cells, which can initiate atherosclerosis. In addition, Ox-LDL induces the formation of foam cells derived from macrophages, which is an important feature at the early stage of atherosclerosis 15.

Many factors affect the uptake and degradation of lipids by cells in atherosclerotic sites. Under physiological condition the level of intracellular cholesterol increases, and various cells can change the expression of lipid uptake receptors by self-regulation to maintain intracellular cholesterol homeostasis, avoid the excessive accumulation of lipids, consequently prevent the formation of foam cells. Inflammatory cytokines can break such negative feedback regulation mediated by intracellular cholesterol level and intake a large amount of lipids which may exceed clearance capacity and eventually turning them into foam cells 16. The presence of lipid-loaded cells is characteristic manifestation of glomerulosclerosis similarly as in atherosclerotic plaques. HMCs have many characteristics similar to vascular smooth muscle cells and can also ingest Ox-LDL; consequently it is widely believed that mesangial cells are closely associated with lipid-mediated renal injury. However, plasma cholesterol levels are not parallel to the degree of glomerulosclerosis. In recent years, studies have revealed that inflammation is a critical factor in kidney damage caused by lipid abnormalities 17.

This study has showed that HMCs ingest Ox-LDL in a physiological state, and inflammatory cytokine IL-1β promotes Ox-LDL uptake, suggesting that inflammation can alter the cholesterol balance of HMCs. Therefore, in the presence of IL-1β, HMCs can take up plenty of Ox-LDL beyond their scavenging capacity, even when intracellular cholesterol levels are high, and eventually become foam cells. These foam cells derived from HMCs can cause glomerulosclerosis 18, indicating that inflammation can affect intracellular cholesterol homeostasis and aggravate renal injury caused by lipids at the cellular level.

### Inflammatory cytokine IL-1β promotes Ox-LDL uptake by HMCs partly via LOX-1 pathway

LOX-1 is a major receptor of Ox-LDL first found in endothelial cells, and Ox-LDL is a crucial risk factor for the formation of atherosclerosis. LOX-1 belongs to the C-type lectin family, is specific to Ox-LDL, and cannot bind to native LDL. After LOX-1 is specifically combined with Ox-LDL, it can activate and damage endothelial cells, increase the expression of adhesion factors and inflammatory cytokines, promote the aggregation and infiltration of monocytes, consume substantial Ox-LDL, and participate in the formation of atherosclerotic plaques, which plays a critical role in atherogenesis 19.

Previous studies confirmed that LOX-1 is mainly expressed in arterial endothelial cells, macrophages, vascular smooth muscle cells, fibroblasts, and other cells 20. The results of this study demonstrated that HMCs cultured in vitro express the LOX-1 receptor. HMCs express a variety of Ox-LDL receptors on their surface, such as scavenger receptor type A, CD36, and SR-BI 21, and six types of scavenger receptors related to Ox-LDL uptake have been identified to date 22. This study showed that IL-1β could promote Ox-LDL uptake by HMCs and LOX-1 receptor blockade reduce Ox-LDL uptake, suggesting that IL-1β stimulated Ox-LDL uptake partly via LOX-1 pathway. Under inflammatory conditions, HMCs can take up a large amount of Ox-LDL via LOX-1 receptor pathway. LOX-1 can induce production of reactive oxygen species (ROS) after intake of Ox-LDL, which can also promote LOX-1 expression in turn. Increased LOX-1 expression can promote Ox-LDL endocytosis, suggesting that there may be a "malignant" cycle involving Ox-LDL-ROS-LOX-1, subsequently activating nuclear factor-κB and other cytokines to trigger an inflammatory response, which plays a crucial role in acceleration of renal injury. Consequently, LOX-1 combined with Ox-LDL not only promotes uptake of lipids, but also induces expression of inflammatory cytokines such as transforming growth factor-β(TGF-β), which stimulate the proliferation of HMCs and accelerate deterioration of kidney disease, suggesting that LOX-1 plays a key contributing role in progression of glomerulosclerosis 23.

### Regulation of LOX-1 expression in HMCs by inflammatory cytokine IL-1β

Various inflammatory cytokines can induce LOX-1 expression and participate in the formation of atherosclerosis. The present study has demonstrated that tumor necrosis factor-α potently induces LOX-1 expression in macrophages 10. Recent study has indicated that interleukin-6 contributes to the upregulation of the LOX-1 gene expression induced by the presence of Ox-LDL in human microvascular endothelial cells 11. Furthmore, it has been demonstrated that Ox‑LDL itself could significantly upregulate LOX‑1 expression in human coronary artery endothelial cells and the self-regulation of LOX‑1 was positively correlated with the concentration of Ox‑LDL 24. Our previous study revealed that Ox-LDL had self-feedback regulation to LOX-1 in HMCs similarly as in endothelial cells. In addition, IL‑1β could increase expression of LOX‑1 in further when co-cultured with Ox‑LDL and consequently promote the uptake of Ox‑LDL by HMCs 25. LOX-1 also plays an important role in the progression of diabetes and chronic kidney disease. Study on diabetic nephropathy reveaved that LOX-1 expression in tubulointerstitial area was significantly correlated with the degree of the tubulointerstitial fibrosis and urinary protein, suggesting that increased expression of LOX-1 may be closely linked to the progression of diabetic nephropathy 26. Previous study on rat model for chronic renal failure after 5/6 nephrectomy demonstrated that LOX-1 was significantly increased in the remnant kidney, accompanied by impaired renal function, suggesting that enhanced renal expression of LOX-1 might play crucial role in the progression of chronic renal failure 27. Recent study in hypercholesterolemic animals showed that Ox-LDL induced up-regulation of TGF-β by LOX-1 signaling, which suggested that LOX-1 may be a potential therapeutic target for renal fibrosis 28. Additionally, Anti-LOX-1 therapy may be an effective method to reverse critical pathogenic elements of nephropathy in dyslipidemia and diabetes 29. In this study, IL-1β promoted expression of LOX-1 significantly, suggesting that the enhancement of Ox-LDL uptake may be mediated by LOX-1 pathway. Therefore, blocking or antagonising the biological effects of LOX-1 may reduce and delay progression of nephropathy.

## Conclusion

The results of this study have indicated that inflammatory cytokine IL-1β increases Ox-LDL uptake, which is partially inhibited by an anti-LOX-1 antibody, suggesting that IL-1β promotes Ox-LDL uptake partly via LOX-1 pathway. Furthermore, IL-1β promotes LOX-1 mRNA and protein expression in a dose- and time-dependent manner. Therefore, inflammation can influence intracellular cholesterol homeostasis through altered expression of receptors affecting lipid uptake and synthesis, consequently activating a variety of cytokines and aggravating renal injury caused by lipids at the cellular level. In conclusion, inflammation is a crucial risk factor for the progression of nephropathy, and anti-LOX-1 therapy can reverse pathological damage and may be a new target for treatment of glomerulosclerosis.

## Figures and Tables

**Table 1:** Changes in the average intracellular fluorescence per unit area in different groups stimulated with IL-1β and blocked with an anti-LOX-1 antibody. * P < 0.05 vs. Control; # P < 0.05 vs. IL-1β group.

Treatment	Average fluorescence value per unit area (n = 3)
Control	6.7 ± 0.23
IL-1β (5 ng/mL)	33.9 **±** 0.51*
IL-1β (5 ng/mL) + Anti-LOX-1	29.8 **±** 0.37^#^

## Abbreviations

FCS: foetal calf serum
HMCs: human glomerular mesangial cells
IL-1β: interleukin-1β
LOX-1: lectin-like Ox-LDL receptor 1
PBS: phosphate-buffered saline
Ox-LDL: oxidised low-density lipoprotein
PCR: polymerase chain reaction
ROS: reactive oxygen species
TGF-β: transforming growth factor-β
